# Ingestion of mycoprotein, pea protein, and their blend support comparable postexercise myofibrillar protein synthesis rates in resistance-trained individuals

**DOI:** 10.1152/ajpendo.00166.2023

**Published:** 2023-08-02

**Authors:** Sam West, Alistair J. Monteyne, Gráinne Whelehan, Ino van der Heijden, Doaa R. Abdelrahman, Andrew J. Murton, Tim J. A. Finnigan, Francis B. Stephens, Benjamin T. Wall

**Affiliations:** ^1^Department of Public Health and Sport Sciences, Faculty of Health and Life Sciences, University of Exeter, Exeter, United Kingdom; ^2^Department of Surgery, University of Texas Medical Branch, Galveston, Texas, United States; ^3^Sealy Center of Aging, University of Texas Medical Branch, Galveston, Texas, United States; ^4^New Era Foods, Yarm, United Kingdom

**Keywords:** muscle protein synthesis, mycoprotein, pea protein, protein blend, resistance exercise

## Abstract

Pea protein is an attractive nonanimal-derived protein source to support dietary protein requirements. However, although high in leucine, a low methionine content has been suggested to limit its anabolic potential. Mycoprotein has a complete amino acid profile which, at least in part, may explain its ability to robustly stimulate myofibrillar protein synthesis (MyoPS) rates. We hypothesized that an inferior postexercise MyoPS response would be seen following ingestion of pea protein compared with mycoprotein, which would be (partially) rescued by blending the two sources. Thirty-three healthy, young [age: 21 ± 1 yr, body mass index (BMI): 24 ± 1 kg·m^−2^] and resistance-trained participants received primed, continuous infusions of l-[*ring*-^2^H_5_]phenylalanine and completed a bout of whole body resistance exercise before ingesting 25 g of protein from mycoprotein (MYC, *n* = 11), pea protein (PEA, *n* = 11), or a blend (39% MYC, 61% PEA) of the two (BLEND, *n* = 11). Blood and muscle samples were taken pre-, 2 h, and 4 h postexercise/protein ingestion to assess postabsorptive and postprandial postexercise myofibrillar protein fractional synthetic rates (FSRs). Protein ingestion increased plasma essential amino acid and leucine concentrations (time effect; *P* < 0.0001), but more rapidly in BLEND and PEA compared with MYC (time × condition interaction; *P* < 0.0001). From similar postabsorptive values (MYC, 0.026 ± 0.008%·h^−1^; PEA, 0.028 ± 0.007%·h^−1^; BLEND, 0.026 ± 0.006%·h^−1^), resistance exercise and protein ingestion increased myofibrillar FSRs (time effect; *P* < 0.0001) over a 4-h postprandial period (MYC, 0.076 ± 0.004%·h^−1^; PEA, 0.087 ± 0.01%·h^−1^; BLEND, 0.085 ± 0.01%·h^−1^), with no differences between groups (all; *P* > 0.05). These data show that all three nonanimal-derived protein sources have utility in supporting postexercise muscle reconditioning.

**NEW & NOTEWORTHY** This study provides evidence that pea protein (PEA), mycoprotein (MYC), and their blend (BLEND) can support postexercise myofibrillar protein synthesis rates following a bout of whole body resistance exercise. Furthermore, these data suggest that a methionine deficiency in pea may not limit its capacity to stimulate an acute increase in muscle protein synthesis (MPS).

## Graphical abstract

**Figure:**
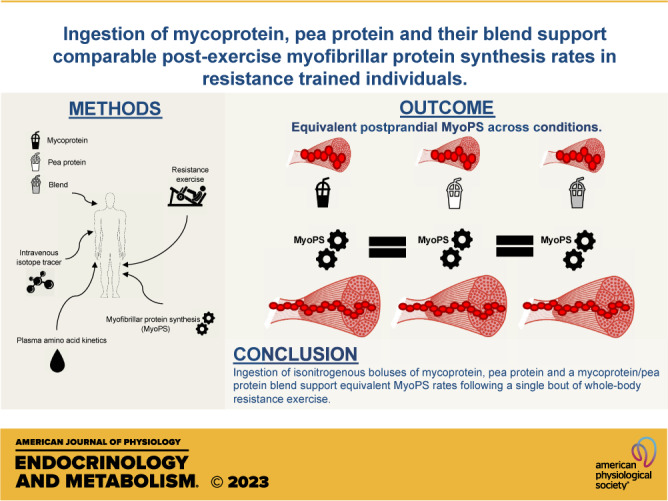

## INTRODUCTION

Dietary protein and muscle contraction increase muscle protein synthesis (MPS) rates, making adequate protein intake and regular exercise essential for the reconditioning of skeletal muscle tissue (1–3). Furthermore, the modality of exercise, particularly the amount of active muscle tissue, can exaggerate the demand for exogenous amino acids, meaning greater servings of protein may be required following whole body compared with single/lower limb exercise (4). Essential amino acids (EAAs) are the main drivers of postprandial MPS rates (5), with the magnitude of the increase thought to be primarily determined by rapid and/or large increases in plasma leucine concentrations (6–8). Animal-derived protein sources are high in EAAs and leucine and, as a result, have frequently been demonstrated to robustly stimulate MPS rates (9–14). However, concerns surrounding the sustainability of (increased) production of animal-based proteins (15) are driving nutritional research to investigate alternative sources (16), particularly for use within sports and exercise nutrition (17).

Plant-based protein sources generally have lower EAA and leucine contents compared with animal-based sources (18, 19) that underpin the suggested, and to some extent observed, inferior capacity to stimulate MPS rates (10, 20). We have demonstrated that mycoprotein, a fungal-derived protein source, is capable of supporting a robust increase in MPS (21, 22), even to a greater extent than milk protein (21), and as a result support longer-term muscle protein turnover (23, 24) and adaptive responses to training (24). This is likely owed to the complete EAA profile and in vivo bioavailability of those amino acids following ingestion (25). Pea protein isolate is a promising alternative protein source on account of its versatility and commercial advancement as a food ingredient (26), cost-effectiveness and sustainable production (27), complete amino acid profile, and high leucine content (18). However, it is low in methionine (<1.6% total protein; World Health Organization (WHO) (18, 28, 29), with single amino acid deficiencies theorized to limit the anabolic capacity of plant proteins (18, 19). Although there are data to demonstrate that pea protein can increase anabolic signaling pathways (30) and support equivalent hypertrophy in response to resistance training compared with whey (31, 32), no studies to date have assessed the acute MPS response to pea protein ingestion.

Blending protein sources has been proposed to mitigate common EAA deficiencies (17, 19). Studies have demonstrated that blending plant- with animal-derived proteins is a viable method for augmenting the postprandial MPS response (33–37), though less work has investigated plant-only protein blends. Two recent studies demonstrate that plant-based protein blends are equally as effective for stimulating MPS rates as animal-derived comparators (milk protein), though both studies provide ≥30 g of protein (likely mitigating any EAA limitation) and data were obtained from resting muscle (38, 39).

In the present work, we first hypothesized that bolus ingestion of mycoprotein (containing 25 g protein) would result in a greater myofibrillar protein synthesis (MyoPS) response following whole body resistance exercise (to maximize systemic muscle amino acid demand and exacerbate any deficiency) compared with an isonitrogenous bolus of pea protein. Second, we hypothesized that blending mycoprotein with pea protein would improve the MyoPS response compared with pea protein alone.

## METHODS

### Participants

Thirty-three resistance-trained, young and healthy individuals volunteered to take part in the present study [age: 21 ± 1 yr, body mass: 75 ± 2 kg, body mass index (BMI): 24 ± 1 kg·m^−2^]. Participants’ characteristics are presented in Table 1. Participants were considered resistance-trained if they were engaged in resistance training >3 times per week for >3 mo before taking part in the study. This population was selected as training status impacts the anabolic response to exercise (40). Therefore, selecting resistance-trained individuals ensured maximal muscle amino acid demand, optimal exercise execution, (more) ecological validity (to exercise training), and an assumed greater homogeneity of responses to exercise. Subjects had not undergone any previous stable isotope tracer protocols in the previous 6 mo ensuring negligible background stable isotope enrichments. Exclusion criteria included any metabolic impairment, cardiovascular complications, or allergies to mycoprotein/edible fungi or environmental molds. Subjects were admitted to the study after being deemed healthy based on blood pressure (<140/90 mmHg), BMI (18–30 kg·m^−2^), and responses to a routine medical health questionnaire. Experimental procedures, potential risks, and the purpose of the study were explained to the participants before obtaining informed written consent. This study was approved by the Sport and Health Sciences ethics committee of the University of Exeter (200325/B/03) in accordance with the Declaration of Helsinki and is registered at ClinicalTrials.Gov (NCT04894747). Recruitment and data collection were carried out in the Nutritional Physiology Research Unit at the University of Exeter between May 2021 and June 2022.

**Table 1.** Participants’ characteristics

	MYC (*n* = 11)	PEA (*n* = 11)	BLEND (*n* = 11)
Sex (M/F)	7/4	9/2	8/3
Age, yr	21 ± 1	20 ± 1	21 ± 1
Height, cm	176 ± 4	175 ± 3	176 ± 3
Weight, kg	75 ± 4	75 ± 3	76 ± 4
BMI, kg·m^-2^	24 ± 1	24 ± 1	24 ± 1
Systolic blood pressure, mmHg	120 ± 3	125 ± 2	123 ± 3
Diastolic blood pressure, mmHg	70 ± 2	70 ± 2	69 ± 2
Fat, % body mass	18 ± 2	14 ± 2	14 ± 2
Lean mass, kg	61 ± 4	64 ± 4	64 ± 4
Leg press 1RM, kg	259 ± 20	247 ± 18	252 ± 22
Leg extension 1RM, kg	101 ± 8	112 ± 8	109 ± 8
Romanian deadlift 1RM, kg	105 ± 9	117 ± 10	110 ± 8
Total exercise volume, kg × rep	12,917 ± 782	12,406 ± 828	14,009 ± 1803
Lower body exercise volume, kg × rep	10,371 ± 667	9,473 ± 712	11,025 ± 820
Upper body exercise volume, kg × rep	2,546 ± 189	2,934 ± 223	2,985 ± 303

Values are represented as means ± SE. No significant differences between groups (all *P* > 0.05). BLEND, mycoprotein/pea blend; BMI, body mass index; MYC, mycoprotein; PEA, pea protein; 1RM, one repetition maximum.

### Pretesting

All participants underwent a pretesting protocol at least 5 days before a single experimental trial. Participants reported to the laboratory to assess body composition, leg strength, and to become familiarized with the exercise protocol to be used during the experimental trial (described in *Resistance Exercise Protocol*). Body composition [body fat (%) and lean mass (kg)] was assessed using Air Displacement Plethysmography (BodPod, Life measurement, Inc. Concord, CA).

### Experimental Protocol

**Figure 1.**
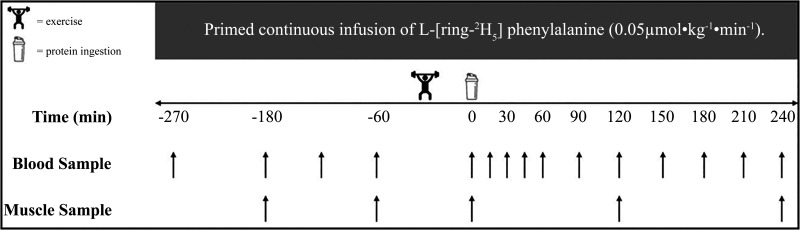
Protocol schematic of the experimental visit.

Participants were randomly assigned to one of three parallel groups and completed a single experimental trial in a double-blind manner. An overview of the experimental protocol can be found in Fig. 1. Participants were asked to avoid vigorous physical activity and alcohol in the 48 h preceding the trial. All participants were provided with a standardized meal to consume as their last food intake ∼10 h before arriving at the laboratory [4.6 MJ (1,110 kcal), 29% energy from fat, 46% energy from carbohydrate, 25% energy from protein].

On the day of the trial, participants arrived at the laboratory at 7:30 AM after the 10-h overnight fast. A Teflon cannula was inserted into an antecubital vein of one arm for the infusion of the stable isotope tracer. Before the infusion was initiated, a baseline venous blood sample was taken from this site to measure background isotopic enrichments. Following the baseline blood sample, the infusion protocol began with a single intravenous priming dose of l-[*ring*-^2^H_5_]phenylalanine (2.12 μmol/kg) (*t* = −270 min). After the priming dose, a continuous tracer infusion was initiated (*t* = −270 min) at a rate of 0.05 μmol·kg^−1^·h^−1^ of l-[*ring*-^2^H_5_]phenylalanine for the duration of the protocol. Once this infusion was in progress, a second Teflon cannula was inserted retrogradely into a dorsal hand vein of the contralateral arm and placed in a heated hand unit (55°C) to allow for subsequent arterialized-venous blood sampling (41). Arterialized venous blood samples were then taken throughout the remainder of the infusion at the following time (*t*) points: −180, −120, −60, 0, 15, 30, 45, 60, 90, 120, 150, 180, 210, and 240 min. Following a 90-min period to allow the achievement of isotopic steady state (22), a baseline muscle biopsy sample was collected (*t* = −180 min), then again at *t* = −60 min from the same leg at least 2 cm distal to the previous incision for the calculation of resting and postabsorptive myofibrillar protein synthesis (MyoPS) rates. Muscle biopsies were sampled from the visual midpoint of the m. vastus lateralis with a modified Bergström suction needle under local anesthetic (2% lidocaine) (42). All biopsy samples were immediately freed from any visible blood, connective, and adipose tissue before being frozen in liquid nitrogen (within 30–60 s) and stored at −80°C until analysis. At −60 min, participants were taken to the research gymnasium adjacent to the laboratory to execute a bout of whole body resistance exercise, as described in *Resistance Exercise Protocol*. Following exercise, a third muscle biopsy was collected from the contralateral leg to that where the initial two biopsies were collected from, before consuming a beverage (details in *Protein Beverage Preparation*) containing 25 g of protein from either mycoprotein (MYC), pea protein (PEA), or a blend of mycoprotein and pea protein (BLEND) (*t* = 0). Each beverage was administered randomly in a double-blind fashion and consumed within an allotted 5-min period. Participants then rested in a semi-supine position for 4 h, with further muscle biopsies collected (from the same leg as biopsy 3) at least 2 cm distal to the previous incisions, 2 and 4 h following beverage consumption to determine postprandial, postexercise MyoPS rates over an early (0–2 h) and later (0–4 h) phase. Following completion of the trial, participants were provided with food and transport home.

Average incorporation times between the collection of biopsies for calculation of resting postabsorptive MyoPS did not differ between groups (MYC, 121 ± 0.3 min; PEA, 122 ± 0.8 min; BLEND 121 ± 0.4 min; *P* > 0.05). Average incorporation times between the collection of biopsies used to calculate 2 h (MYC, 121 ± 0.5 min; PEA, 122 ± 0.9 min; BLEND 122 ± 0.5 min; *P* > 0.05) and 4 h (MYC, 241 ± 0.5 min; PEA, 241 ± 0.4 min; BLEND 241 ± 0.7 min *P* > 0.05) postprandial MyoPS rates also did not differ between groups.

### Resistance Exercise Protocol

During the pretesting visit, three repetition max (RM) was assessed to estimate 1RM for leg press, leg extension, and Romanian deadlift (RDL) exercises (43). 3RM, rather than 1RM, was selected as an accurate approach to predict 10RM to minimize safety risks (43). Strength testing began with a brief warm-up with a low weight on each exercise. Thereafter, participants attempted a self-selected weight for 3RM. Weight was increased for each subsequent attempt with final 3RM being accepted as the last weight lifted correctly before a failed attempt (±5 kg from failed attempt). 10RM was then calculated as 70% of estimated 1RM.

Once 3RM testing had finished, participants rested for ∼5 min and were then asked to complete one set (∼10 repetitions) at the calculated 70% 1RM for familiarization and verification purposes. Participants were then familiarized with the upper body exercises consisting of cable chest fly (pectoral), reverse dumbbell fly (posterior deltoid, rhomboids), straight arm cable pull-down (latissimus dorsi), and lateral dumbbell raise (medial deltoids). The weight assigned for the upper body exercises was estimated based on body mass of the participant by multiplying body mass by a correction factor for each exercise (chest fly, 1.12/0.6; reverse fly, 0.22/0.15; lateral raise, 0.2/0.15; straight arm pulldown, 1/0.8; male/female, respectively) aiming to ensure failure after ∼10 repetitions. The exercises included were selected to target the whole body to provide a comprehensive and maximal stimulus of a large volume of muscle mass while also minimizing risks associated with exercising while attached to intravenous infusion lines (e.g., elbow flexion). As well as increasing ecological validity, this nature of exercise maximizes postexercise systemic amino acid demand and therefore has been suggested to alter the postexercise protein requirements to maximize the MPS response (4).

For the experimental trial, exercises were performed in the following order: chest fly, reverse dumbbell fly, straight arm cable pulldown, lateral dumbbell raise, leg press, leg extension, RDL. Upper body exercises were performed as supersets (chest fly/rear deltoid dumbbell fly; straight arm cable pulldown/lateral dumbbell raise). Participants performed four sets of each exercise separated by 90-s rest. The first set of each exercise was performed as 10 repetitions at 75% of calculated 10RM. Participants were instructed to work to failure on the remaining three sets at their 10RM. For subsequent sets, the weight was increased when participants were able to perform >12 repetitions and decreased when participants were unable to perform 8 repetitions. Verbal encouragement was provided throughout. Average time spent performing the entire exercise protocol did not differ between conditions (MYC, 53 ± 1 min; PEA, 57 ± 2 min; BLEND, 54 ± 2 min; *P* > 0.05). Average time between the completion of exercise and the next muscle biopsy was 20 ± 1 min.

### Protein Beverage Preparation

Freeze-dried mycoprotein was produced and provided by Marlow Foods Ltd., Quorn Foods, Stokesley, UK. Mycoprotein was produced as previously described (44). Pea protein was produced and supplied by Corsucra Ltd., Warcoing, Belgium. The mycoprotein and pea blend was produced by mixing the freeze-dried powders (39% MYC, 61% PEA). All protein sources were independently analyzed (Premier Analytical Services, UK) for energy, macronutrient content, and amino acid composition, the details of which are displayed in Table 2.

**Table 2.** The nutritional composition of the experimental beverages [(46.7 g of mycoprotein, 31.7 g of pea protein and 36.9 g of mycoprotein/pea protein blend (39/61%)]

	MYC	PEA	BLEND
Macronutrients			
Protein, g	25	25	25
Carbohydrate, g	2.8	1.0	1.6
Fat, g	3.5	1.7	1.9
Fiber, g	11.3	0.8	4.7
Energy, kcal	165	121	133
Energy, kJ	692	512	560
Amino acid content, g			
Alanine	1.4	1.0	1.1
Arginine	1.5	2.0	1.8
Asparagine	2.1	2.7	2.5
Cysteine	0.2	0.2	0.5
Glutamine	2.7	4.0	3.4
Glycine	1.0	1.0	1.0
Histidine	0.5	0.6	0.5
Isoleucine	1.0	1.1	1.0
Leucine	1.7	1.9	1.7
Lysine	1.7	1.7	1.6
Methionine	0.4	0.2	0.3
Phenylalanine	1.0	1.3	1.2
Proline	1.0	1.0	1.0
Serine	1.1	1.3	1.2
Threonine	1.2	0.9	1.0
Tryptophan	0.4	0.2	0.3
Tyrosine	0.8	0.9	0.8
Valine	1.2	1.1	1.1
EAA	10.5	10.9	10.5
BCAA	3.9	4.1	3.9

Protein content (g) is calculated from the sum of the amino acids measured after complete hydrolysis. BCAA, branched-chain amino acids; BLEND, mycoprotein/pea protein blend; EAA, essential amino acids; MYC, mycoprotein; PEA, pea protein.

The evening before the experimental trial the powdered protein sources were assimilated with 400 mL of water and 40 mL of energy-free flavoring (Clearwater, FL), blended for ∼2 min and refrigerated overnight (440 mL of final fluid volume). Drinks were enriched (2%) with l-[*ring*-^2^H_5_]phenylalanine to maintain systemic isotopic precursor steady state following protein ingestion (21). During the experimental trial, once participants had consumed the drink, an additional 100 mL water were added to “rinse” the bottle and ensure that all the protein had been consumed. Double blinding of the drinks was achieved by having a separate researcher from those carrying out the experimental trial visits prepare the drinks in an opaque bottle. The drinks were matched for protein content (25 g) requiring 47.7, 31.7, and 36.9 g of MYC, PEA, or BLEND powders, respectively.

### Blood Sample Collection and Analyses

Ten milliliters of arterialized-venous (with the exception of the baseline sample which was a venous collection) blood were collected into a syringe at each time point. Five milliliters of that sample were added to EDTA-containing tubes (BD vacutainer LH; BD Diagnostics, Nu-Care) and centrifuged for 10 min at 4,000 rpm at 4°C. The plasma supernatant was then removed, aliquoted, and stored at −80°C for later analyses. The remaining 5 mL of blood were added to additional vacutainers (BD vacutainers SST II, BD Diagnostics, Nu-Care) and left upright to clot at room temperature for 30 min and then centrifuged for 10 min at 4,000 rpm at 4°C. The serum supernatant was then removed, aliquoted, and stored at −80°C for future analyses.

Serum insulin concentrations were measured using a commercially available ELISA kit (DRG Insulin ELISA, EIA-2935, DRG International Inc., Springfield, NJ). Plasma l-[*ring*-^2^H_5_]phenylalanine enrichments (MPEs) and concentrations of glycine, phenylalanine, leucine, valine, isoleucine, lysine, histidine, glutamic acid, methionine, proline, serine, threonine, tyrosine, and alanine were determined in *tert*-butyldimethylsilyl derivatives by GC-MS with electron impact ionization (Agilent, Santa Clara, CA) as described previously (45). Briefly, to prepare samples for GC-MS, 10 μL of 2 mM nor-leucine was added as an internal standard to 450 μL plasma and deproteinized on ice with 450 μL of 15% 5-sulfosalicylic acid. Samples were then vortexed and centrifuged at 4,000 rpm for 10 min at 4°C. The supernatant was then loaded onto cation exchange columns. Columns were then filled with ddH_2_O, followed by 6 mL of 0.5 M acetic acid, and then washed once more with ddH_2_O, with the columns allowed to drain between each step. The amino acids were then eluted with 2 mL of 6 M ammonia hydroxide (NH_4_OH). The eluate was dried using a Speed-Vac for 8 h at 60°C before undergoing derivatization (as described in the *Muscle Tissue Analyses*) for muscle.

### Muscle Tissue Analyses

Myofibrillar protein extractions were performed as previously described (46). The process was carried out with ∼50 mg of muscle tissue, which was homogenized using a mechanical glass pestle in a glass tube in homogenization buffer [in mM: 50 Tris·HCl pH 7.4, 1 EDTA, 1 EGTA, 10 b-glycerophosphate salt, 50 NaF, and 0.5 activated Na_3_VO_4_; (Sigma-Aldrich Company Ltd., Poole, UK)] with a complete protease inhibitor cocktail tablet [1 tablet per 50 mL of buffer; Roche, Burgess Hill, UK]). The homogenate was transferred into a clean 2-mL Eppendorf and centrifuged at 2,200 *g* for 10 min at 4°C. The supernatant (sarcoplasmic fraction) was aliquoted and stored at −80°C for subsequent analysis. The remaining pellet was then washed in 500 μL of homogenization buffer and centrifuged again at 700 *g* for 10 min at 4°C and the resultant supernatant was discarded. The remaining protein portion (myofibrillar and collagen) (47) was then solubilized in 750 μL of 0.3 M sodium hydroxide and heated at 50°C for 30 min and centrifuged at 10,000 *g* for 5 min at 4°C. The supernatant (myofibrillar fraction) was then aliquoted into a new 2-mL Eppendorf and precipitated in 500 μL of 1 M perchloric acid. These samples were centrifuged at 700 *g* for 10 min at 4°C and the resultant supernatant was discarded. The remaining myofibrillar pellet was washed in 1 mL of 70% ethanol and centrifuged at 700 *g* for 5 min at 4°C before the ethanol was removed. This step was repeated once more before the amino acids were then hydrolyzed by adding 2 mL of 6 M hydrochloric acid and heating at 110°C for 24 h. Once hydrolyzed, the amino acids were then dried on a heating block (110°C) for 24 h. Samples were then reconstituted in 1.5 mL of 25% acetic acid and pipetted into the cation exchange column. The Eppendorf was then rinsed with another 1.5 mL of 25% acetic acid. The columns were then eluted with 2 mL of 6 M NH_4_OH into a 2-mL Eppendorf and the eluate dried in a Speed-Vac for 8 h at 60°C. Samples were cleaned by adding 1 mL of ddH_2_O and 1 mL of 0.1% formic acid in acetonitrile and centrifuged at 10,000 *g* for 3 min at 4°C. The supernatant was aliquoted into a new Eppendorf and dried in the Speed-Vac for 5 h at 60°C. To derivatize the muscle sample, 50 μL of MTBSTFA + 1% *tert-*butyldimethylchlorosilane and 50 μL of acetonitrile were added to the dry samples, vortexed, and heated at 95°C for 45 min (48). The samples were analyzed by GC-MS (7890 GC coupled with a 5975 MSD; Agilent Technologies) in triplicate using electron impact ionization and selected ion monitoring for measurement of isotope abundance (49). One microliter of the sample was injected in splitless mode (injector temperature: 280°C). Peaks were resolved using an HP5-MS 30 m × 0.25 mm inner diameter (ID) × 0.25 μm capillary column (Agilent). Helium was used as the carrier gas at 1.2 mL/min constant flow rate. The temperature ramp was set from 80°C to 245°C at 11 °C/min, then to 280°C at 40 °C/min (49). Selected ion recording conditions were used to monitor fragments *m*/*z* 237 and 239 for the *m* + 3 and *m* + 5 fragments of phenylalanine-bound protein and *m*/*z* 336 and 341 for the *m* + 0 and *m* + 5 fragments of the phenylalanine-free fraction. A single linear standard curve from mixtures of known *m* + 5/*m* + 0 ratios for l-[*ring*-^2^H_5_]phenylalanine was used to determine the enrichments of the protein-bound samples using the *m* + 5/*m* + 3 ratio.

### Calculations

The fractional synthetic rates (FSRs) of myofibrillar proteins were calculated using the standard precursor-product equation (45):

$$\text{FSR}(\%\cdot h-1)=\left[\Delta E_{\text{p}}/E_{\text{precursor}}\times t\right]\times 100$$

where Δ*E*_p_ is the increment in protein-bound l-[*ring*-^2^H_5_]phenylalanine in myofibrillar protein between two muscle biopsies, *E*_precursor_ is the average l-[*ring*-^2^H_5_]phenylalanine enrichment in the plasma precursor pool over time, and *t* indicates the time (h) between two muscle biopsies.

### Statistical Analysis

A two-sided power analysis with expected effect sizes estimated from previous research (10, 21) revealed that *n* = 10 in each group was sufficient to detect expected differences in postprandial, postexercise MyoPS rates between protein conditions (MYC vs. PEA vs. BLEND) when using a repeated-measures analysis of variance (ANOVA) (*P* < 0.05, Power 80%, *f* = 0.67; G∗power v.3.1.9.2). Factoring in a 20% drop-out rate, 36 participants were therefore recruited for the study. The study recruited 36 participants with 3 drop-outs, therefore data is presented for *n* = 33. Statistical significance was set at *P* < 0.05. All calculations were performed on GraphPad 7.1. Participants’ characteristics, total work done, and background l-[*ring*-^2^H_5_]phenylalanine enrichments were analyzed using independent samples *t* tests. Differences in serum insulin concentrations, plasma amino acid concentrations, plasma tracer enrichments, and myofibrillar l-[*ring*-^2^H_5_]phenylalanine enrichments were compared using two-way [group (MYC vs. PEA vs. BLEND) × time] repeated-measures ANOVA. Separate two-way ANOVAs were performed on postabsorptive and postexercise postprandial plasma l-[*ring*-^2^H_5_]phenylalanine enrichments. MyoPS rates were calculated as FSRs and analyzed using a two-way (group × time) ANOVA. Total postprandial insulin and amino acid availabilities were calculated as incremental area under the curve (iAUC) with a baseline set as an average of *t* = −180, −120, −60, and 0.

## RESULTS

### Participants’ Characteristics

No differences in body mass, height, BMI, body fat percentage, lean mass, or strength (leg press, leg extension, RDL [1RM]) were found between groups (all *P* > 0.05; Table 1). Total work (repetition × weight) performed across all exercises did not differ between conditions (MYC, 12,917 ± 782 kg; PEA, 12,406 ± 828 kg; BLEND, 14,009 ± 1083 kg; *P* > 0.05). When separated for total work performed for the upper body exercises, work done also did not differ between groups (MYC, 2546 ± 189 kg; PEA, 2934 ± 223 kg; BLEND, 2985 ± 303 kg; *P* > 0.05). Similarly, total work performed for the lower body exercises did not differ between groups (MYC, 10,371 ± 667 kg; PEA, 9,473 ± 712 kg; BLEND, 11,025 ± 820 kg; *P* > 0.05).

### Serum Insulin and Plasma Amino Acid Concentrations

**Figure 2.**
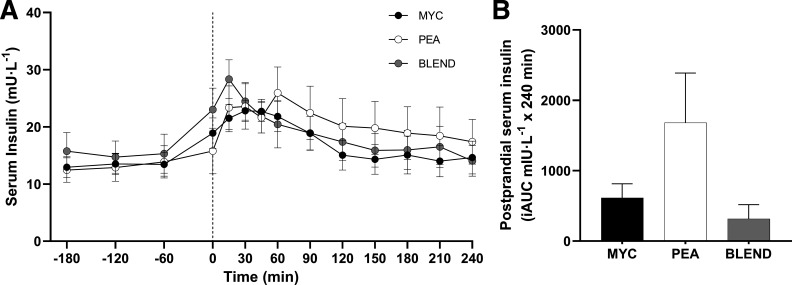
Time course (*A*) and incremental area under the curve (iAUC; *B*) (calculated as above postaborptive values) of serum insulin concentrations for a 3-h postabsorptive period (time course only) and a 4-h postprandial period in healthy resistance-trained men. The dashed vertical line represents drink consumption [46.7 g of mycoprotein containing 25 g of protein (MYC; *n* = 11), 31.7 g of pea protein containing 25 g of protein (PEA; *n =* 11), or 36.9 g of mycoprotein/pea protein blend (39/61%) containing 25 g of protein (BLEND; *n* = 11)], following a bout of whole body resistance exercise. Time course data were analyzed using a two-way repeated-measures ANOVA (group × time) with Sidak post hoc tests used to detect differences at individual time points. iAUC data were analyzed using a one-way ANOVA. Time effect; *P* < 0.0001. Group effect; *P* > 0.05. Group × time interaction; *P* > 0.05. Values are represented as means ± SE.

All serum insulin and plasma amino acid concentrations are presented for *n* = 33 (MYC, 11; PEA, 11; BLEND, 11). Serum insulin concentrations over the time course of the experiment are presented in Fig. 2. Fasting serum insulin concentrations were similar between groups. The ingestion of protein significantly increased serum insulin concentrations (time effect; *P* < 0.001) to a similar extent between groups (*P* > 0.05). Accordingly, postprandial serum insulin iAUC did not differ between groups (*P* > 0.05).

**Figure 3.**
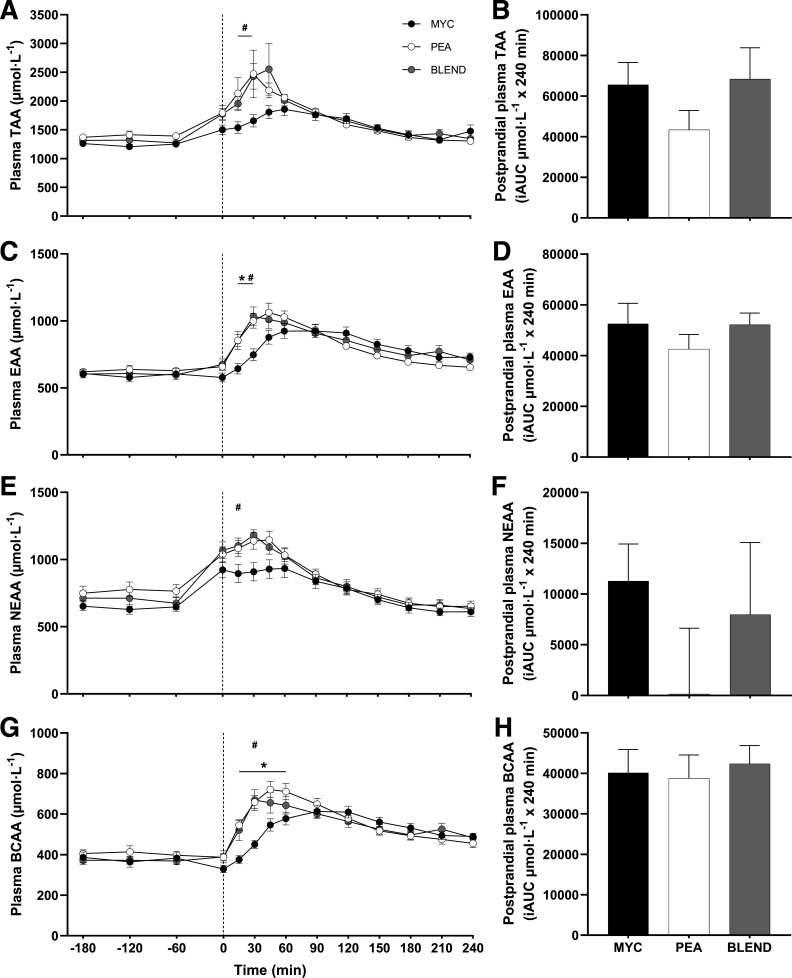
Time course and incremental area under the curve (iAUC; calculated as above postaborptive values) of plasma total amino acids (TAA) (*A* and *B*), essential amino acids (EAA) (*C* and *D*), nonessential amino acids (NEAA) (*E* and *F*), and branched-chain amino acids (BCAA) (*G* and *H*) over a 3-h postabsorptive period (time course only) and 4-h postprandial period in healthy resistance-trained men. The dashed vertical line represents drink consumption [46.7 g of mycoprotein containing 25 g of protein (MYC; *n* = 11), 31.7 g of pea protein containing 25 g of protein (PEA; *n =* 11), or 36.9 g of mycoprotein/pea protein blend (39/61%) containing 25 g of protein (BLEND; *n* = 11)], following whole body resistance exercise. Time course data were analyzed using a two-way repeated-measures ANOVA (group × time) with Sidak post hoc tests used to detect differences at individual time points. iAUC data were analyzed using a one-way ANOVA. *Individual differences between MYC and PEA at that time point and a difference between conditions on the bar graphs (*P* < 0.05). #Individual differences between MYC and BLEND at that time point (*P* < 0.05). Time effect; all *P* < 0.0001. Group effect; *P* > 0.05. Time × group interaction; all *P* < 0.0001. Values are represented as means ± SE.

**Figure 4.**
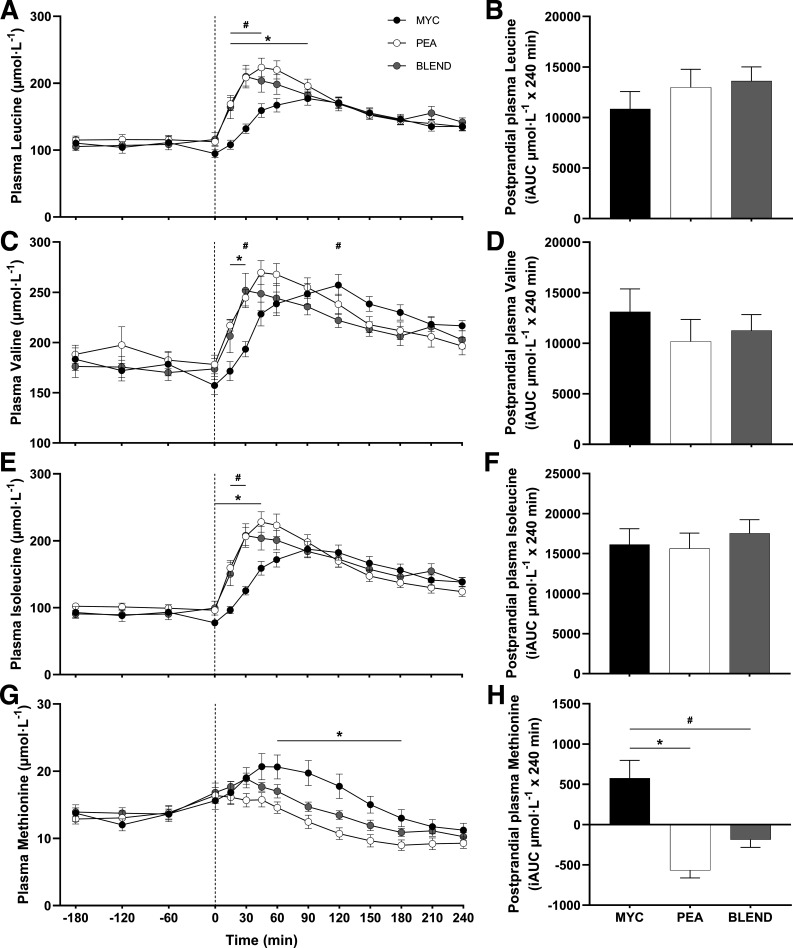
Time course and incremental area under the curve (iAUC; calculated as above postaborptive values) of plasma leucine (*A* and *B*), valine (*C* and *D*), isoleucine (*E* and *F*), and methionine (*G* and *H*) over a 3-h postabsorptive period (time course only) and 4-h postprandial period in healthy resistance-trained men. The dashed vertical line represents drink consumption [46.7 g of mycoprotein containing 25 g of protein (MYC; *n* = 11), 31.7 g of pea protein containing 25 g of protein (PEA; *n =* 11), or 36.9 g of mycoprotein/pea protein blend (39/61%) containing 25 g of protein (BLEND; *n* = 11)], following whole body resistance exercise. Time course data were analyzed using a two-way repeated-measures ANOVA (group × time) with Sidak post hoc tests used to detect differences at individual time points. iAUC data were analyzed using a one-way ANOVA. *Individual differences between MYC and PEA at that time point and a difference between conditions on the bar graphs (*P* < 0.05). #Individual differences between MYC and BLEND at that time point (*P* < 0.05). Time effect; all *P* < 0.0001. Group effect; all *P* > 0.05, with exception of leucine *P* < 0.05. Time × group interaction; all *P* < 0.0001. Values are represented as means ± SE.

**Figure 5.**
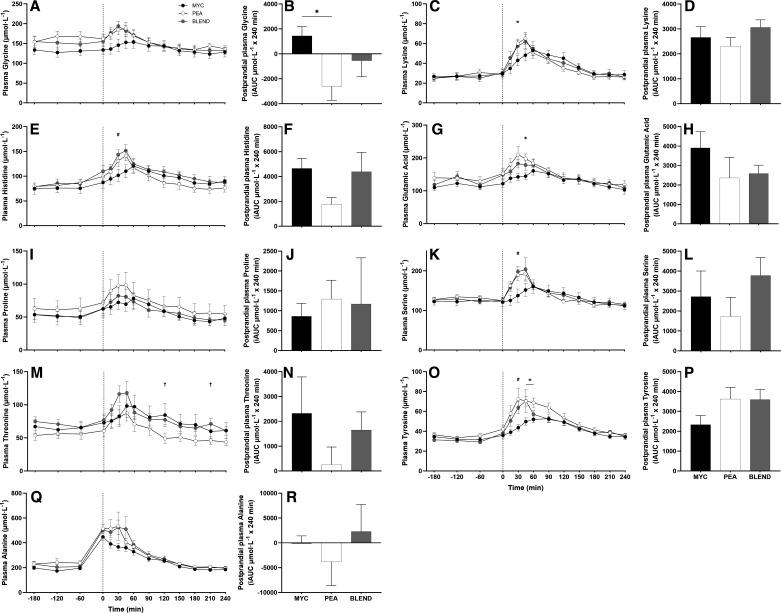
Time course and incremental area under the curve (iAUC; calculated as above postaborptive values) of plasma glycine (*A* and *B*), lysine (*C* and *D*), histidine (*E* and *F*) glutamic acid (*G* and *H*), proline (*I* and *J*), serine (*K* and *L*), threonine (*M* and *N*), tyrosine (*O* and *P*), and alanine (*Q* and *R*) over a 3-h postabsorptive period (time course only) and 4-h postprandial period in healthy resistance-trained men. The dashed vertical line represents drink consumption [46.7 g of mycoprotein containing 25 g of protein (MYC; *n* = 11), 31.7 g of pea protein containing 25 g of protein (PEA; *n =* 11), or 36.9 g of mycoprotein/pea protein blend (39/61%) containing 25 g of protein (BLEND; *n* = 11)], following whole body resistance exercise. Time course data were analyzed using a two-way repeated-measures ANOVA (group × time) with Sidak post hoc tests used to detect differences at individual time points. iAUC data were analyzed using a one-way ANOVA. *Individual differences between MYC and PEA at that time point and a difference between conditions on the bar graphs (*P* < 0.05). #Individual differences between MYC and BLEND at that time point (*P* < 0.05). †Individual differences between PEA and BLEND at that time point (*P* < 0.05). Time effect; all *P* < 0.0001. Group effect; all *P* > 0.05. Time × group interaction; all *P* < 0.05 with exception of proline and alanine. Values are represented as means ± SE.

Plasma total (TAA), essential (EAA), branched-chain (BCAA), nonessential (NEAA), and individual amino acid concentrations over the time course of the experiment are presented in Figs. 3, 4, and 5. All plasma amino acid concentrations changed over time (time effects; *P* < 0.0001). This increase occurred following protein ingestion for all amino acids with the exception of alanine, which showed a large increase before protein ingestion and consequent to exercise. Plasma concentrations of TAA, NEAA, EAA, and BCAA displayed a more rapid increase following ingestion of PEA and BLEND compared with MYC (time × group interactions; all *P* < 0.0001). Plasma leucine concentrations peaked more rapidly in the PEA and BLEND conditions compared with MYC (time × group interactions; all *P* < 0.0001) with greater concentrations detected at 15, 30, 45, and 60 with PEA, and 15 and 30 min with BLEND, compared with MYC. No differences were observed at any time point between PEA and BLEND for all amino acids (*P* > 0.05), except for threonine, where concentrations were elevated at 120 and 210 min in BLEND compared with PEA (*P* > 0.05). Plasma methionine concentrations decreased following ingestion of PEA and increased following ingestion of MYC and BLEND (time × group interaction; *P* < 0.0001). Accordingly, plasma methionine concentrations were elevated at 60, 90, 120, 150, and 180 min in MYC compared with PEA. Methionine concentrations were elevated during the postprandial period compared with PEA, and lower than MYC, though not statistically different from either condition at any timepoint (*P* > 0.05).

Total postprandial plasma amino acid availabilities, calculated as iAUC, are also displayed in Figs. 3, 4, and 5 for all amino acid parameters (inset graphs). Greater plasma methionine availability was observed in the MYC compared with PEA and BLEND conditions (*P* < 0.0001). Furthermore, methionine availability was greater in the BLEND compared with PEA condition (*P* < 0.0001). There was also greater plasma availability of glycine in the MYC compared with PEA conditions (*P* < 0.05). Postprandial plasma amino acid availability for all other plasma amino acid outcomes did not differ between groups (*P* > 0.05).

### Plasma and Skeletal Muscle Tracer Analysis

The samples from one participant from the MYC group were excluded from tracer analyses due to insufficient muscle tissue. Therefore, all data presented for plasma and muscle l-[*ring*-^2^H_5_]phenylalanine are for *n* = 32 (*n* = 10 MYC, *n* = 11 PEA, *n* = 11 BLEND).

**Figure 6.**
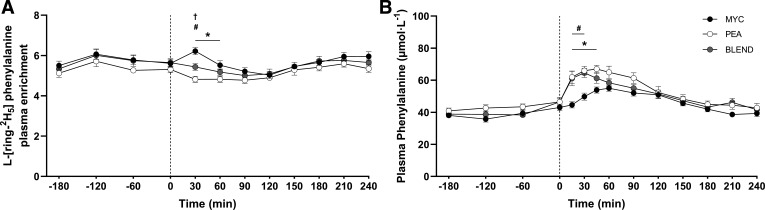
Time course of plasma l-[*ring*-^2^H_5_]phenylalanine enrichments (*A*) and plasma phenylalanine concentrations (*B*) during the experimental trial over a 3-h postabsorptive period and 4-h postprandial period in healthy resistance-trained men. The dashed vertical line represents drink consumption [46.7 g of mycoprotein containing 25 g of protein (MYC; *n* = 11), 31.7 g of pea protein containing 25 g of protein (PEA; *n =* 11), or 36.9 g of mycoprotein/pea protein blend (39/61%) containing 25 g of protein (BLEND; *n* = 11)], following whole body resistance exercise. Time course data were analyzed using a two-way repeated-measures ANOVA (group × time) with Sidak post hoc tests used to detect differences at individual time points. *Individual differences between MYC and PEA at that time point and a difference between conditions on the bar graphs (*P* < 0.05). #Individual differences between MYC and BLEND at that time point (*P* < 0.05). †Individual differences between PEA and BLEND at that time point (*P* < 0.05). Time effect; both *P* < 0.0001. Group effect; phenylalanine concentrations *P* < 0.05, l-[*ring*-^2^H_5_]phenylalanine enrichments *P* > 0.05. Time × group interaction; all *P* < 0.05. Values are represented as means ± SE.

The time course of plasma l-[*ring*-^2^H_5_]phenylalanine enrichments are displayed in Fig. 6. Plasma l-[*ring*-^2^H_5_]phenylalanine enrichments changed over time (time effect; *P* < 0.0001) and to a different extent between groups (time × group interaction; *P* < 0.05). Plasma l-[*ring*-^2^H_5_]phenylalanine enrichments were elevated between 30 and 60 min in the MYC compared with PEA (*P* < 0.0001) and at 30 min compared with BLEND (*P* < 0.05). Plasma l-[*ring*-^2^H_5_]phenylalanine enrichments were higher in the BLEND compared with PEA conditions at the 30-min time point (*P* > 0.05). Isotopic steady state had been regained in all groups by 120 min.

Myofibrillar protein-bound l-[*ring*-^2^H_5_]phenylalanine enrichments were equivalent between groups at baseline (*P* > 0.05). Myofibrillar protein-bound l-[*ring*-^2^H_5_]phenylalanine enrichments increased and to the same extent in each group (time effect; *P* < 0.0001, time × group interaction; *P* > 0.05) during the postabsorptive period (0.0038 ± 0.001 to 0.0067 ± 0.002 in MYC; 0.0039 ± 0.001 to 0.0069 ± 0.002 in PEA; 0.0039 ± 0.001 to 0.0067 ± 0.001 in BLEND). Exercise increased myofibrillar protein-bound l-[*ring*-^2^H_5_]phenylalanine enrichments to the same extent between groups (time effect; *P* < 0.0001, time × group interaction; *P* > 0.05) (0.0067 ± 0.002 to 0.0107 ± 0.001 in MYC; 0.0069 ± 0.002 to 0.0125 ± 0.003 in PEA; 0.0067 ± 0.001 to 0.0135 ± 0.002 in BLEND). Protein ingestion further increased myofibrillar protein-bound l-[*ring*-^2^H_5_]phenylalanine enrichments at 2 h (0.0107 ± 0.001 to 0.0191 ± 0.002 in MYC; 0.0125 ± 0.003 to 0.0211 ± 0.003 in PEA; 0.0135 ± 0.002 to 0.0230 ± 0.002 in BLEND) and 4 h (0.0107 ± 0.001 to 0.0277 ± 0.002 in MYC; 0.0125 ± 0.003 to 0.0303 ± 0.004 in PEA; 0.0135 ± 0.002 to 0.0311 ± 0.003 in BLEND) (time effect; *P* < 0.0001) with no differences between groups at any time point (*P* > 0.05).

**Figure 7.**
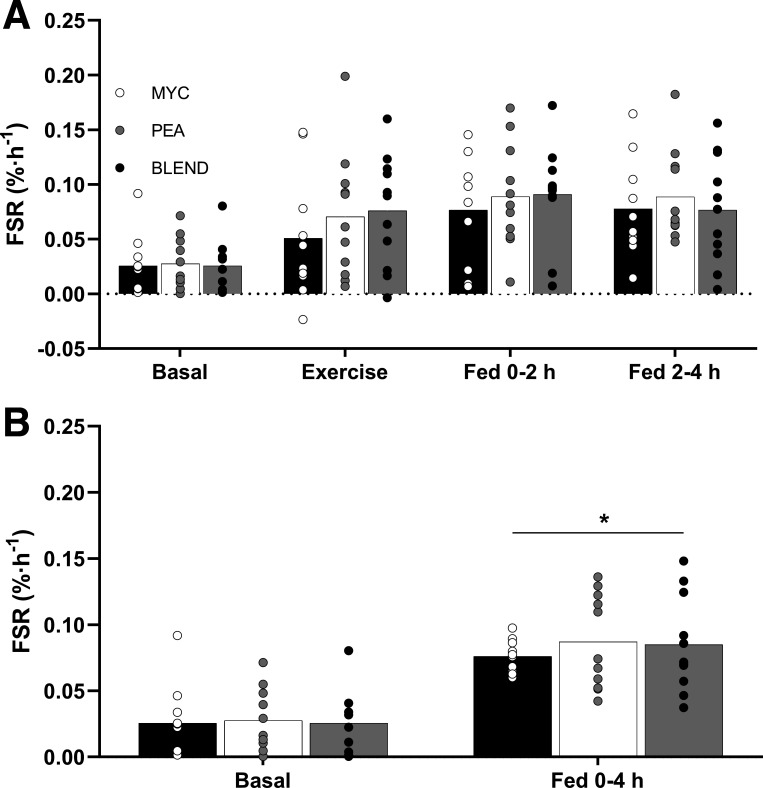
Myofibrillar protein fractional synthetic rates (FSRs) calculated using the plasma l-[*ring*-^2^H_5_]phenylalanine precursor pool for a postabsorptive (basal), exercise, and temporal postprandial periods (0–2 h and 2–4 h) (*A*) and a total 4-h postprandial period (*B*) for mycoprotein (MYC), pea protein (PEA), and mycoprotein/pea protein blend (BLEND) conditions. Postprandial state represents a 4-h period following drink consumption [46.7 g of mycoprotein containing 25 g of protein (MYC; *n* = 11), 31.7 g of pea protein containing 25 g of protein (PEA; *n =* 11), or 36.9 g of mycoprotein/pea protein blend (39/61%) containing 25 g of protein (BLEND; *n* = 11)], following whole body resistance exercise. Data were analyzed using a two-way (group × time) ANOVA. *A significant difference between basal and postexercise postprandial conditions (group × time interaction; *P* < 0.0001). Time effect; *P* < 0.0001. Group effect; *P* > 0.05. Values are represented as means ± SE.

Myofibrillar FSRs were calculated using the average plasma l-[*ring*-^2^H_5_]phenylalanine enrichments during the prandial period of interest as the precursor pool (Fig. 7). Postabsorptive myofibrillar FSRs were similar between groups (MYC, 0.026 ± 0.008%·h^−1^; PEA, 0.028 ± 0.007%·h^−1^; BLEND, 0.026 ± 0.006%·h^−1^; *P* > 0.05). Whole body resistance exercise increased myofibrillar FSRs during exercise (time effect; *P* < 0.05) to a similar extent in each group (MYC, 0.051 ± 0.017%·h^−1^; PEA, 0.071 ± 0.017%·h^−1^; BLEND, 0.076 ± 0.015%·h^−1^; time × group interaction; *P* > 0.05). Myofibrillar FSRs remained elevated compared with postabsorptive rates during the early (MYC, 0.077 ± 0.015%·h^−1^; PEA, 0.089 ± 0.014%·h^−1^; BLEND, 0.091 ± 0.013%·h^−1^; *P* < 0.05) and late (MYC, 0.078 ± 0.014; PEA, 0.089 ± 0.012; BLEND, 0.077 ± 0.014; time effect *P* < 0.05) postprandial period, with no differences between groups observed at either timepoint (time × group interaction; *P* > 0.05), and no differences between early and late periods irrespective of group (*P* > 0.05). Accordingly, there were no differences between groups in myofibrillar FSRs over the entire 4-h postprandial window (MYC, 0.076 ± 0.004%·h^−1^; PEA, 0.087 ± 0.01%·h^−1^; BLEND, 0.085 ± 0.01%·h^−1^; time × group interaction; *P* > 0.05).

## DISCUSSION

The present study compared postexercise myofibrillar protein synthesis (MyoPS) rates following ingestion of isonitrogenous boluses of mycoprotein, pea protein, and a mycoprotein/pea protein blend. We hypothesized that ingestion of mycoprotein would support greater rates of postexercise MyoPS over a 4-h postprandial period compared with pea protein, owing to its more complete amino acid profile. Furthermore, we hypothesized that blending the two sources would correct for the amino acid deficiencies and rescue the lower response seen with pea protein. However, contrary to our hypotheses, we observed similar postexercise MyoPS rates following ingestion of all three protein sources.

Due to increasing concerns surrounding the sustainability of animal protein production, research is investigating the application of nonanimal proteins to support postexercise skeletal muscle remodeling (17). We have previously demonstrated that mycoprotein is a bioavailable protein source capable of supporting acute (21, 22) and intermediate MyoPS (23, 24), and, resultantly, longer-term muscle hypertrophy (24). Pea protein is widely available in commercial (sports) nutrition products (26), due to its versatility and cost-effective/sustainable production (27), relatively balanced amino acid profile, and high leucine content (18) [although a low methionine content is a proposed limitation to its anabolic potential (16, 19)]. However, data on the MyoPS response from studies in humans are lacking. In agreement with previous work (30), we demonstrate a rapid increase and high availability of plasma leucine, branched-chain amino acid (BCAA), and (most) EAA concentrations following pea protein ingestion (see Figs. 3, 4, and 5). This postprandial aminoacidemia was more rapid when compared with mycoprotein ingestion, though overall bioavailability throughout the experiment was equivalent. Unsurprisingly, methionine availability over the 4-h postprandial period was significantly lower following pea protein ingestion compared with mycoprotein (Fig. 4). In fact, plasma methionine concentrations decreased following pea protein ingestion resulting in a negative iAUC, suggesting that postprandial plasma methionine clearance was greater than exogenous appearance. Though this was likely exaggerated by the whole body nature of the exercise performed, the same observation has been reported previously following plant protein ingestion, even at rest (39). To illustrate, the difference in methionine content between the mycoprotein and pea beverages in the present study was ∼67%, yet the difference in plasma methionine 4-h iAUC was ∼200%. Similarly, Pinckaers et al. (39) observed ∼160% differences in 5-h plasma methionine availability between milk protein and a plant protein blend (7.5 g pea) despite only a ∼55% difference in methionine content between drinks. These data imply that small amounts of certain AAs in pea/plant protein display a lower less bioaccessibility and, therefore, are less efficiently digested and/or absorbed into peripheral circulation. Indeed, the methionine-containing albumin fraction of pea protein has been shown to exhibit poor digestibility (50). This suggests that considerations of amino acid composition alone may not inform how much of a given amino acid will become available in circulation.

The common (often single) amino acid deficiencies reported for various plant-based proteins have led researchers to suggest that blending different sources may be a strategy to overcome the theoretical substrate limitation to optimal MPS rates (17, 19), particularly postexercise (4). Although a sports nutrition strategy, blending is also commonplace in the habitual diet as meals provide protein of differing amino acid compositions from a variety of sources. We aimed to mitigate the amino acid deficiencies (particularly methionine) within pea protein by blending it with mycoprotein. This approach increased the methionine content [though still considered deficient; (28)] and, upon ingestion, attenuated the negative postprandial plasma methionine availability (iAUC) compared with pea protein ingestion. Blending the two protein sources also favorably modulated plasma leucine kinetics [rapidity (MYC, 90 min; BLEND, 30 min) and magnitude (MYC, 177 µmol·L^−1^; BLEND, 210 µmol·L^−1^) of appearance] compared with mycoprotein which, based on current consensus (6, 7, 10, 13, 51), should result in an earlier and/or greater increase in MyoPS. To pick apart these potential effects of leucine signaling and substrate limitation we assessed temporal (2 and 4 h) post- (whole body) exercise MyoPS rates. However, irrespective of the diverse postprandial plasma amino acid kinetics across all three conditions, we report equivalent postexercise MyoPS in the early, late, and total postprandial periods (see Fig. 7). This corroborates previous data comparing plant- versus animal-based sources (52) and protein blends (36, 38, 39), which also demonstrated equivalent stimulation of MyoPS despite differences in plasma methionine (and other amino acids) availabilities. Furthermore, these data now support a wider body of evidence showing a disconnect between plasma leucine/amino acid kinetics and postprandial MyoPS rates when comparing protein sources (21, 22, 37–39, 52–57). Therefore, while blending is clearly an effective strategy to modulate and/or improve amino acid composition and postprandial plasma amino acid kinetics, current evidence implies this does not necessarily appreciably affect the MyoPS response. Although previous work has implied that protein blending can increase MyoPS, bringing parity with a high-quality (animal derived) control (33–35, 38, 39), few have included a single source plant control condition to demonstrate unequivocally. Indeed, those that have observed equivalent rates of MyoPS across all conditions (36, 37), in congruence with the present work.

There are various aspects to contemplate as to why we did not observe a requirement for blending within the design of the current work. Although we used a lower protein dose (25 g vs. 30 g) than previous comparable studies, combined with whole body exercise to maximize the possibility of substrate limitation (4), the dose of 25 g may still be adequate to mitigate any amino acid shortcomings (though negative postprandial methionine availability suggests otherwise). It is necessary, therefore, for future work to assess at what protein dose (if ever) amino acid deficiencies curtail the stimulation of MyoPS. This would be more obviously pertinent to populations where protein intake and/or diet quality may be compromised, or where the anabolic response to protein ingestion may be impaired (58). Second, while the whole body exercise likely increased demand for exogenous amino acids across all tissues, the contractile stimulus will also have independently stimulated MyoPS at the site of interest (vastus lateralis). Evidence of the intense and exhaustive nature of the exercise stimulus is demonstrated by a rise of plasma alanine concentrations following exercise, but before protein ingestion (see Fig. 5). This is likely attributable to increased alanine efflux from muscle, to buffer excess pyruvate production in muscle and provide substrate for increased rates of gluconeogenesis to support blood glucose homeostasis during increased demands of exercise (59, 60). Therefore, it is plausible such a maximal contractile stimulus obfuscated any nutrient-related regulatory differences between groups. In addition, exercise increases muscle protein breakdown (2) and improves amino acid recycling (3, 61), potentially mitigating any specific amino acid deficiency, at least in the short term. However, we cannot confirm an improvement in amino acid recycling as we did not apply a 3-pool model. This implies our pragmatic 4-h postprandial period may have been too brief for substrate limitation to become restrictive to the synthesis of new tissue. In support, bolus ingestion of 21 g of protein that was devoid of phenylalanine and tyrosine robustly increased mixed muscle protein synthesis over 3 h, though admittedly there was no “complete” protein used for comparison in this study (62). There are currently no data assessing the role of single amino acid deficiencies and MyoPS over periods of days or weeks. Nonetheless, diets devoid (63) or low (64, 65) of methionine result in a negative nitrogen balance over 7–24 days. Given the limitations of nitrogen balance studies (66, 67), research assessing muscle protein turnover/accretion over a period of days/weeks to assess at what stage single amino acid deficiencies become rate limiting is warranted.

Beyond single amino acid deficiencies, it is interesting to consider if multiple amino acid deficiencies are more consequential with respect to limiting acute postprandial (postexercise) MyoPS rates. It has been shown that the ingestion of BCAAs or branched chain keto acids alone (68, 69), EAAs (70), or a small bolus of protein enriched with EAAs (71), are all capable of stimulating an early (1–2 h) increase in MyoPS. However, these are evidently deficient in multiple amino acid substrates and, accordingly, are unable to support more sustained (3–5 h) increases in MyoPS compared with complete protein sources (68–72). Nevertheless, data are not entirely consistent, as the ingestion of low doses of EAAs + leucine stimulated an equivalent postexercise MyoPS rates over a 4-h postprandial period compared with whey protein (73, 74). Clearly, to understand the role (or necessity) of protein blending, more work is needed to elucidate the relationship between amino acid deficiencies and acute muscle remodeling. Future research will need to investigate the role of multiple and single (specific) amino acid deficiencies to assess under what circumstances (e.g., dose, time period) it becomes limiting to muscle reconditioning.

In conclusion, ingestion of isonitrogenous boluses of mycoprotein, pea protein, and a mycoprotein/pea protein blend support equivalent MyoPS rates following a single bout of whole body resistance exercise. Therefore, while protein blending is a theoretically attractive strategy to correct for lower amino acid content and/or bioavailability, we do not provide evidence within this design that is necessary for optimally stimulating postexercise MyoPS rates. Nonetheless, these data are the first to demonstrate pea protein as an effective protein source to support the postexercise MyoPS response.

## DATA AVAILABILITY

Data described in the manuscript may be made available upon request, pending application.

## GRANTS

This study was part of a studentship funded by Marlow Foods LTD with B. T. Wall as PI and grant holder. S. West and G. Whelehan are supported by a studentship in collaboration with Marlow Foods LTD. A. J. Murton and D. R. Abdelrahman are supported in part by a grant from the National Institute of Aging P30-AG024832.

## DISCLOSURES

T.J.A. Finnigan was an employee of Marlow Foods; B.T. Wall, A.J. Monteyne, and F.B. Stephens are employees of the University of Exeter. The private partners have contributed to the project through regular discussion. Aside from those aforementioned, the authors report no conflicts of interest.

## AUTHOR CONTRIBUTIONS

S.W., A.J.M., T.J.A.F., F.B.S., and B.T.W. conceived and designed research; S.W., G.W., and I.v.d.H. performed experiments; S.W., D.R.A., and A.J.M. analyzed data; S.W., F.B.S., and B.T.W. interpreted results of experiments; S.W. prepared figures; S.W. and B.T.W. drafted manuscript; S.W., A.J.M., G.W., I.v.d.H., F.B.S., and B.T.W. edited and revised manuscript; S.W., A.J.M., G.W., I.v.d.H., D.R.A., A.J.M., T.J.F., F.B.S., and B.T.W. approved final version of manuscript.
